# Regulation of Inflammatory and Proliferative Pathways by Fotemustine and Dexamethasone in Endometriosis

**DOI:** 10.3390/ijms22115998

**Published:** 2021-06-01

**Authors:** Tiziana Genovese, Rosalba Siracusa, Ramona D’Amico, Marika Cordaro, Alessio Filippo Peritore, Enrico Gugliandolo, Rosalia Crupi, Angela Trovato Salinaro, Emanuela Raffone, Daniela Impellizzeri, Salvatore Cuzzocrea, Roberta Fusco, Rosanna Di Paola

**Affiliations:** 1 Department of Chemical, Biological, Pharmaceutical and Environmental Sciences, University of Messina, 98168 Messina, Italy; tgenovese@unime.it (T.G.); rsiracusa@unime.it (R.S.); rdamico@unime.it (R.D.); aperitore@unime.it (A.F.P.); rfusco@unime.it (R.F.); dipaolar@unime.it (R.D.P.); 2 Department of Biomedical, Dental and Morphological and Functional Imaging, University of Messina, 98125 Messina, Italy; cordarom@unime.it; 3 Department of Veterinary Sciences, University of Messina, 98168 Messina, Italy; egugliandolo@unime.it (E.G.); rcrupi@unime.it (R.C.); 4 Department of Biomedical and Biotechnological Sciences, University of Catania, 95124 Catania, Italy; trovato@unict.it; 5 Multi-Specialist Institute Rizzo, Torregrotta, 98043 Messina, Italy; emanuelaraffone@virgilio.it

**Keywords:** endometriosis, inflammation, pathways

## Abstract

Endometriosis is a common disease. Its pathogenesis still remains uncertain, but it is clear that cell proliferation, apoptosis and chronic inflammation play an important role in its development. This paper aimed to investigate the anti-proliferative and anti-inflammatory effects of a combined therapy with fotemustine and dexamethasone. Endometriosis was induced by intraperitoneal injections of uterine fragments from donor animals to recipient animals. Next, the pathology was allowed to develop for 7 days. On the seventh day, fotemustine was administered once and dexamethasone was administered daily for the next 7 days. On Day 14, the animals were sacrificed, and peritoneal fluids and lesions were explanted. In order to evaluate the gastrointestinal side effects of the drugs, stomachs were harvested as well. The combined therapy of fotemustine and dexamethasone reduced the proinflammatory mediator levels in the peritoneal fluid and reduced the lesions’ area and diameter. In particular, fotemustine and dexamethasone administration reduced the heterogeneous development of endometrial stroma and glands (histological analysis of lesions) and hyperproliferation of endometriotic cells (immunohistochemical analysis of Ki67 and Western blot analysis of PCNA) through the mitogen-activated protein kinase (MAPK) signaling pathway. Combined fotemustine and dexamethasone therapy showed anti-inflammatory effects by inducing the synthesis of anti-inflammatory mediators at the transcriptional and post-transcriptional levels (Western blot analysis of NFκB, COX-2 and PGE2 expression). Fotemustine and dexamethasone administration had anti-apoptotic activity, restoring the impaired mechanism (TUNEL assay and Western blot analysis of Bax and Bcl-2). Moreover, no gastric disfunction was detected (histological analysis of stomachs). Thus, our data showed that the combined therapy of fotemustine and dexamethasone reduced endometriosis-induced inflammation, hyperproliferation and apoptosis resistance.

## 1. Introduction

Endometriosis is an invasive disease which affects women of reproductive age. The hallmarks of the pathology are growth of the endometrial stroma and glands outside the uterine cavity. The symptoms described by the patients are severe dysmenorrhea, chronic pelvic pain and infertility. This pathology, in fact, largely affects women of reproductive age. Although many theories about the etiology of endometriosis have been described, its pathogenesis remains uncertain. Sampson’s hypothesis of retrograde menstruation is the most widely accepted. According to this theory, endometriosis derives from fragments of the endometrial glands. During menstruation, detached endometrial glands, cells and debris, by retrograde movement, reach the peritoneum to implant and grow. Other theories are the theory of Mullerian remnants and coelomic metaplasia. The first theory proposes that cellular fragments from embryonic Mullerian ducts evolve into endometriotic foci at the beginning of puberty through the influence of sex hormones. The second theory proposes that endometrial and peritoneal cells would both have their origin from the coelomic epithelium, thus, during the disease, healthy peritoneal tissue would transform into ectopic endometrium [1]. Even if the theories differ regarding the origin of the pathology, all agree that endometriotic lesions’ growth is supported by the inflammatory microenvironment, hyperproliferation and impaired apoptosis [2,3]. The endometrium constitutes the functionalis or superficialis layer, which undergoes cyclic remodeling, and the basalis layer, which is stable. In particular, during the menstrual cycle, the functionalis layer is tightly regulated by hormones: it becomes hypoxic and necrotic, and is shed. Menstrual fragments are composed of both living and necrotic cells [4,5].

Thus, cell death has an important role in homeostasis maintenance and removing excess or dysfunctional cells. A balance between cell proliferation and apoptosis to maintain differentiated tissue is a crucial protective mechanism against endometriosis development [3,6]. Many studies have shown that apoptosis maintains endometrial homeostasis by the menstrual cycle removing aged cells from the functional layer of the endometrium [7]. A reduced apoptosis rate has been described in the endometrial cells of patients [8,9,10], indicating that cells that arrive in the peritoneal cavity have a higher survival rate [8,11].

These migrating endometriotic stromal cells are producers of inflammatory mediators such as prostaglandins, cytokines and metalloproteinases, which are secreted in the surrounding environment [12]. Elevated levels of interleukin (IL)-6, vascular endothelial growth factor (VEGF), IL-1β, tumor necrosis factor alpha (TNF-α), IL2 and prostaglandin (PG)E2 in the serum and peritoneal fluid of women could be used as non-invasive markers of the pathology [13]. The pro-inflammatory environment, in turn, is overactivated and overexpresses crucial transcriptional factors and the mediators involved in endometriosis development [14]. In particular, IL-1β and TNF-α promote the increase of PGE2 levels in stromal and epithelial endometrial cells in endometriotic foci by inducing cyclooxygenase (COX)-2 overexpression, nuclear factor kappa-light-chain-enhancer of activated B cells (NFκB) activation and the mitogen-activated protein (MAP) kinases pathway [15,16]. Thus, treatments that counteract these molecular characteristics would be useful for disease management.

Fotemustine (FT) is an alkylating agent belonging to the nitrosurea family. It has anti-proliferative activities and is used for the treatment of glioma and brain metastasis. Unfortunately, FT has important toxicities, including gastric disfunction and myelosuppression [17,18]. The side effects of FT are progressive, dose-dependent and cumulative [19]. Here, we evaluated the effects of 10 mg/kg of FT as a single dose, which is one-fourth of the maximum tolerated dose, and combined it with 0.1 mg/kg/day i.p. dexamethasone (DM) to reduce the inflammation in the peritoneal microenvironment.

Previous studies, in fact, have demonstrated that glucocorticoid has anti-inflammatory effects related to the inhibition of the IL-1β [20], TNF-α [21], COX-2 [22], inducible nitric oxide synthase (iNOS) [23], NFκB [24] and MAPK [25] pathways.

In this paper, we evaluated the combined effects of low doses of FT and DM in a rat model of endometriosis to ensure higher efficacy in suppressing endometriotic lesions’ growth and inflammation.

## 2. Results

### 2.1. Effect of FT+DM on the Inflammatory Microenvironment

**Figure 1:**
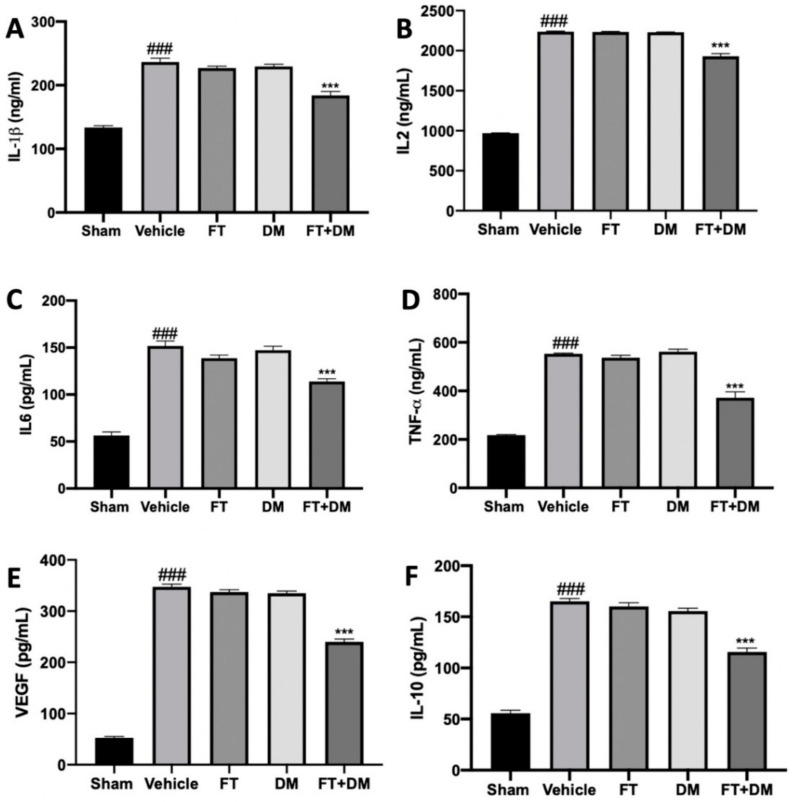
FT+DM administration reduced the levels of cytokines: IL-1β (**A**), IL2 (**B**), IL6 (**C**), TNF-α (**D**), VEGF (**E**) and IL-10 (**F**) levels in peritoneal fluid. A *p*-value of less than 0.05 was considered significant: *** *p* < 0.001 vs. vehicle, ### *p* < 0.001 vs. sham.

Several papers have described the changes in the peritoneal district as an important parameter for the establishment and development of endometriosis. Thus, inflammatory cytokine levels in peritoneal fluid were detected. As compared with the sham-treated rats, increased levels of IL-1β (Figure 1A), IL-2 (Figure 1B), IL-6 (Figure 1C), TNF-α (Figure 1D), VEGF (Figure 1E) and IL-10 (Figure 1F) were detected in vehicle-treated rats.

Peritoneal fluids harvested from FT- and DM-administered rats did not show any difference in cytokine levels as compared with the vehicle group. FT+DM combined administration was able to reduce IL-1β (Figure 1A), IL-2 (Figure 1B), IL-6 (Figure 1C), TNF-α (Figure 1D), VEGF (Figure 1E) and IL-10 (Figure 1F) expression in the peritoneal fluids.

### 2.2. Effect of FT+DM on Endometriotic Lesions

**Figure 2:**
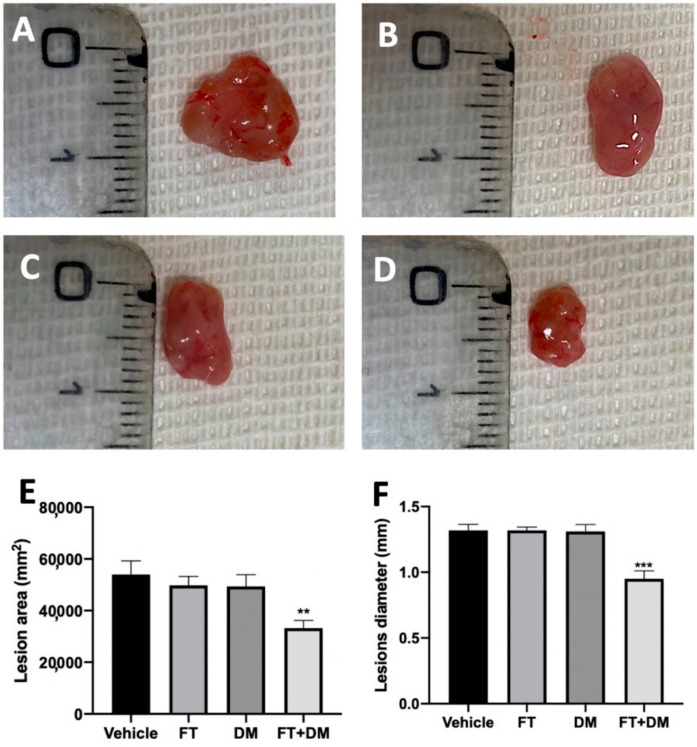
FT+DM administration reduced the size of endometrioc foci (macroscopic analysis): vehicle (**A**), FT (**B**), DM (**C**), FT+DM (**D**), lesion area (**E**), lesion diameter (**F**). A *p*-value of less than 0.05 was considered significant: ** *p* < 0.01 vs. vehicle, *** *p* < 0.001 vs. vehicle.

The changes in the peritoneal fluids also reflect the development of the endometriotic foci. No implants were detected in sham rats, while the vehicle, FT, DM and FT+DM groups did not differ in cyst numbers. Macroscopic analysis of the lesions showed a significant reduction in the lesions’ area (Figure 2E) and diameter (Figure 2F) in the FT+DM group (Figure 2D) as compared with the vehicle-treated group (Figure 2A). Lesions harvested from the FT (Figure 2B) and DM (Figure 2C) groups did not differ from the vehicle-treated group for area (Figure 2E) and diameter (Figure 2F).

### 2.3. Effect of FT+DM on Histological Score and Hyperproliferation

**Figure 3:**
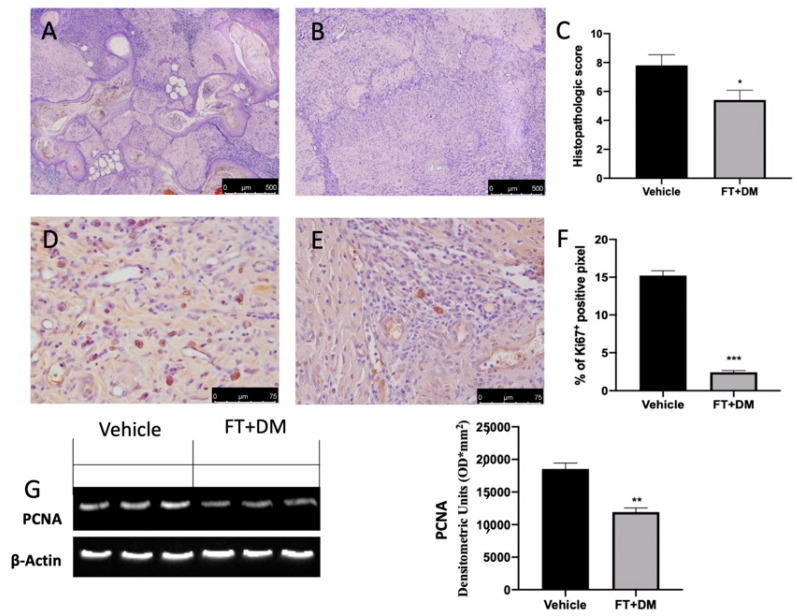
FT+DM administration reduced histological score, hyperproliferation and apoptosis. Histological analysis: vehicle (**A**); FT+DM (**B**). Histological score (**C**). Immunohistochemical analysis of Ki67: vehicle (**D**); FT+DM (**E**). Graphical quantification of KI67 expression (**F**). Western blot analysis of PCNA (**G**). A *p*-value of less than 0.05 was considered significant: * *p* < 0.05, ** *p* < 0.01 vs. vehicle, *** *p* < 0.001 vs. vehicle.

Histological analysis of the lesions harvested from vehicle-treated rats displayed endometrial-type glands and stromal structure (Figure 3A,C). FT+DM administration reduced the histopathologic score (Figure 3B,C).

An essential characteristic of endometriosis is hyperproliferation. Immunohistochemical analysis showed elevated Ki67 expression in lesions collected from vehicle-treated rats (Figure 3D,F), which were reduced by FT+DM administration (Figure 3E,F). Western blot analysis showed elevated PCNA expression in the FT+DM group as compared with the vehicle group (Figure 3G).

### 2.4. Effect of FT+DM on the MAPK Pathway

**Figure 4:**
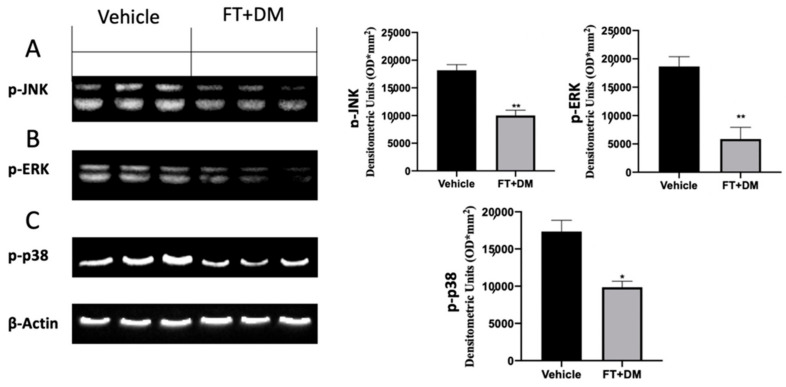
FT+DM administration reduced MAPK activation: Western blot analysis of p-JNK (**A**), p-ERK (**B**) and p-p38 (**C**). A *p*-value of less than 0.05 was considered significant: * *p* < 0.05 vs. vehicle, ** *p* < 0.01 vs. vehicle.

The MAPK pathway is significantly upregulated in endometriotic lesions. Western blot analysis confirmed increased expression of p-JNK (Figure 4A), p-ERK (Figure 4B) and p-p38 (Figure 4C) in the vehicle group. FT+DM administration reduced p-JNK (Figure 4A), p-ERK (Figure 4B) and p-p38 (Figure 4C) expression.

### 2.5. Effect of FT+DM on NFκB and COX-2 Expression

**Figure 5:**
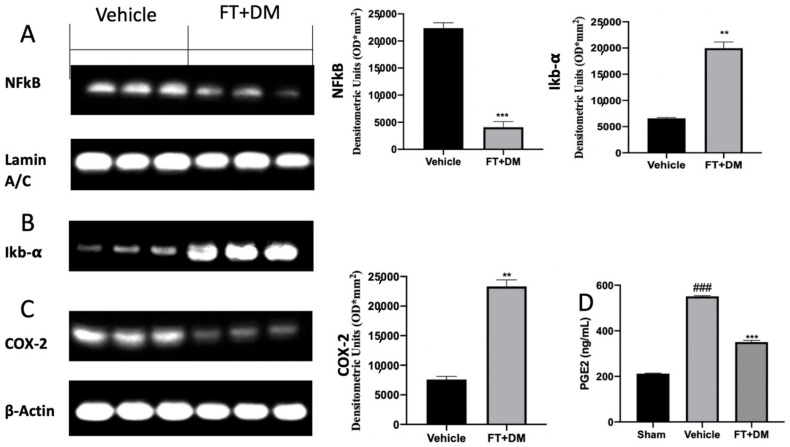
FT+DM administration reduced inflammation: Western blot analysis of NFκB (**A**), IκB-α (**B**) COX-2 (**C**) and PGE2 expression levels (**D**). A *p*-value of less than 0.05 was considered significant: ** *p* < 0.01 vs. vehicle, *** *p* < 0.001 vs. vehicle, ### *p* < 0.001 vs. sham.

The activation of several inflammatory pathways has been described as influencing the progression of endometriosis. Western blot analysis showed high NFκB expression in the nucleus and low Iκb-α expression in the cytosol of samples harvested from vehicle-treated rats, while FT+DM administration significantly reduced NFκB expression in the nucleus (Figure 5A) and increased Iκb-α expression in the cytosol (Figure 5B). Additionally, increased COX-2 (Figure 5C) and PGE2 (Figure 5D) expression was detected in vehicle-treated rats, and FT+DM administration was able to reduce both levels (Figure 5C,D).

### 2.6. Effect of FT+DM on Apoptosis

**Figure 6:**
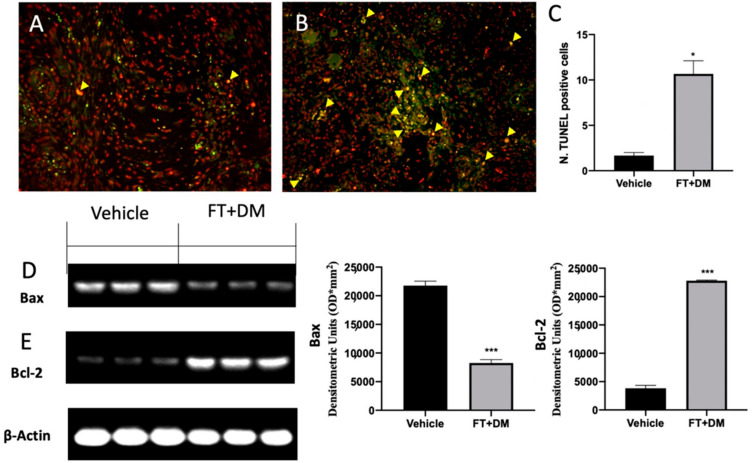
FT+DM administration reduced apoptosis. Histological analysis and TUNEL assay (40× magnification; 75 μm scale bar): vehicle (**A**); FT+DM (**B**). Number of TUNEL positive cells (**C**). Western blot analysis of Bax (**D**) and Bcl-2 (**E**). A *p*-value of less than 0.05 was considered significant: * *p* < 0.05 vs. vehicle, *** *p* < 0.001 vs. vehicle.

Impaired apoptosis is one of the key features of endometriosis. The TUNEL assay showed an increased number of cells undergoing apoptosis in the FT+DM group (Figure 6B,C) as compared with the vehicle group (Figure 6A,C). Western blot analysis displayed low Bax and elevated Bcl-2 expression in samples from vehicle-treated rats, while FT+DM treatment increased Bax (Figure 6D) and reduced Bcl-2 (Figure 6E).

### 2.7. Effect of FT+DM on Gastric Homeostasis

**Figure 7:**
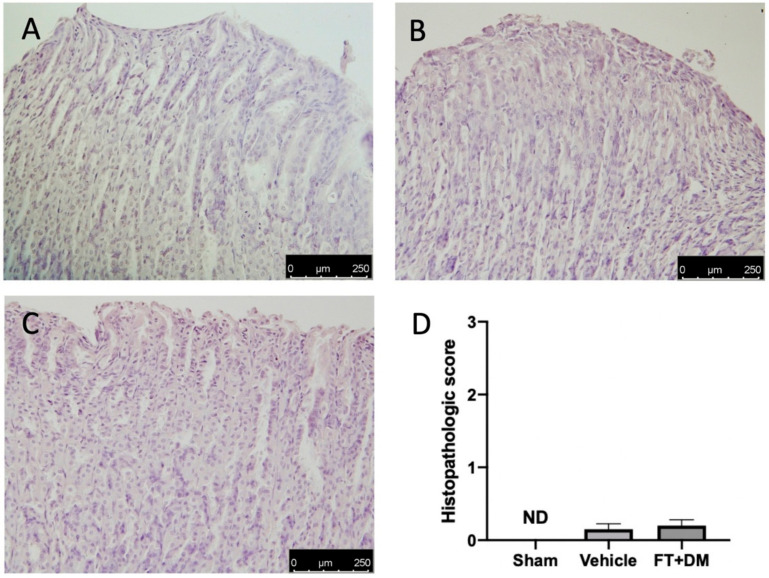
FT+DM administration did not show any gastric injury. Histological analysis: sham (**A**), vehicle (**B**) and FT+DM (**C**); histological score (**D**). Scale bar 250 μm.

Gastric injury is one of the best described side effects of FT and DM. Histological analysis of stomachs harvested from vehicle-treated rats did not show any significant gastric damage (Figure 7A,D) as compared with the sham-treated animals (Figure 7B,D); the FT+DM group did not show any gastric dysfunction (Figure 7C,D).

## 3. Discussion

This paper evaluated the combined effect of FT and DM on endometriosis, evaluating the association between anti-proliferative and anti-inflammatory approaches. The interaction between the local inflammatory environment and an endometriotic lesion has been described [26]. In the past, the relationship between inflammation and endometriosis was seen in infertile women, in whom intraperitoneal inflammation was observed [27], and was thought to be partially due to retrograde menstruation [28,29]. These findings suggested that inflammation may be involved in the pathogenesis of endometriosis.

Endometriotic implants secrete VEGF and proinflammatory cytokines such as TNF-α, IL-6, IL-1, IL-2 and IL-10, among others [30]. This combination of secretions in the peritoneal fluid promotes an angiogenic and proliferative environment that promotes the progression of endometriosis. Single administration of FT or DM at the tested doses did not modify inflammatory molecule secretion, while FT+DM administration significantly reduced this cocktail of inflammatory mediators in the peritoneal fluid. Accordingly, in animals treated with the FT+DM combination, in the reduced inflamed peritoneal microenvironment, endometriotic lesions with reduced area and diameter were found, compared with those found in animals treated with single administration of FT and DM.

Our data clearly showed that single administration of a low dose of FT or DM did not affect the development and progression of endometriosis, while their co-administration would be helpful to counteract disease progression.

Thus, following these results, we investigated the molecular pathway managed by FT+DM administration in the explanted lesions.

The histological and immunohistochemical analysis showed modified cyst morphology, cell proliferation and apoptosis. Histologically, FT+DM reduced the heterogeneous development of endometrial stroma and glands, surrounded by zones of pronounced inflammation caused by the incorrect location of the endometrium. In line with the literature, immunohistochemical staining displayed an increased number of proliferating stromal and epithelial cells in the lesions. The mean cell proliferative index was significantly larger in those with endometriosis as compared with controls [31]. Many papers have shown the importance of cell proliferation during endometriosis, evaluating the expression of Ki-67 and PCNA proteins. Ki-67 is a nucleolar and nuclear protein that provides crucial information on cellular information disorders [32]. It is expressed during all phases of cell cycle except for the G1 phase. PCNA is expressed during the DNA synthesis phase in the nuclei of cells and is a marker of proliferation as well [33]. It is used for grading different benign lesions and different neoplasms with proliferative behavior [34]. FT+DM administration significantly reduced cell proliferation, as shown by Ki-67 immunostaining and PCNA Western blot analysis. This result can be explained by considering that chemotherapeutic agents have more efficacy when applied to rapidly growing cells [35,36]. Experimental data obtained from in vivo and in vitro studies and human clinical trials have shown that resting cells are less sensitive to anti-cancer drugs than proliferating cells [37,38]. The mediators that stimulate cell proliferation are managed by many intracellular signaling cascades. The MAPK pathway is strongly perturbed in endometriotic lesions, and it has an important role in the proliferative mechanism [39,40]. In stromal endometriotic cells, upregulation of MAP kinases have been described in response to pro-inflammatory mediators [41,42,43].

FT+DM administration reduced the phosphorylation of JNK, ERK and p-38, showing anti-proliferative and anti-inflammatory effects. In particular, p38 acted on many nuclear or cytosolic substrates to induce cellular responses, including the synthesis of inflammatory mediators at the transcriptional and posttranscriptional levels [44]. Among these, NFκB is one of the most important [45,46,47,48,49]. The NFκB pathway is often activated by inflammatory stimuli [50,51,52,53]. In physiological condition, NFκB complexes, constituted by NFκB dimers and Iκb protein inhibitors, are located in the cytoplasm. In this bound form, NFκB is inactive and unable to translocate in the nucleus and to bind DNA. In answer to inflammatory stimuli, Iκb inhibitory proteins are degraded, and thus NFκB is released and free to translocate into the nucleus [54,55,56]. In particular, FT+DM administration increased the expression of the IκB-α protein, promoting NFκB retention in the cytoplasm and its permanence in the inactive state. The activation of the NFκB pathway increased COX-2 and prostaglandin expression [14,26]. The COX-2 isoenzyme is normally synthetized at low levels, while it is over-expressed under inflammatory conditions [57]. COX-2 overexpression catalyzes arachidonic acid conversion in PGH2, and is then converted to PGF2 and PGE2 by PGF and PGE synthase respectively. In peritoneal fluids of women affected by endometriosis, increased PGE2 concentrations have been found [58]. PGE2 is a well-known anti-apoptotic mediator that impairs programmed cell death. Together with hyperproliferation, apoptosis is an impaired mechanism during endometriosis. It is significantly activated in the secretory stage and is downregulated in the proliferative stage of the menstrual phase in the normal endometrium [59,60,61,62,63]. By contrast, apoptosis decreases during the luteal and proliferative stage in refluxed endometrial tissues. FT+DM administration significantly reduced COX-2 overexpression and PGE2 levels. Additionally, it was able to partially restore the apoptosis mechanism in the endometriotic lesions.

Our data showed that the combined administration of FT and DM substantially reduced the pro-inflammatory environment that contributes to development of endometriotic lesions, acting on hyperproliferation and apoptosis resistance. Addionally, our study demonstrated that the low doses tested did not show any gastrointestinal side effects, which are among the main side effects of FT [17,18] and DM [64,65].

## 4. Materials and Methods

### 4.1. Animals

Female Sprague–Dawley rats (Envigo, Milan, Italy) were used in this research. The University of Messina Review Board for animal care (OPBA) approved the study. All animal experiments complied with the new Italian regulations (D.Lgs 2014/26), EU regulations (EU Directive 2010/63) and the ARRIVE guidelines.

### 4.2. Experimental Protocol

Animals were randomly divided into 2 groups (donor or recipient), and endometriosis was established as already described [14]. To stimulate similar estrogen levels, donor rats were intraperitoneally injected with 10 IU pregnant mare serum gonadotropin and euthanized 41 h later by CO_2_ asphyxia. A midline incision was performed, and the uterus was removed and minced with scissors. Tissue from all donors was pooled, and the recipient animals were injected intraperitoneally with the equivalent of tissue from one uterus in 500 μL of PBS along the midventral line. Endometriosis was allowed to develop for 7 days.

### 4.3. Experimental Groups

Rats were randomized and assigned to the following groups:

(1) Vehicle group: Rats were subjected to experimental endometriosis as described above, and the vehicle (saline) was administered by gavage on the seventh day and for the next 7 days.
(2) FM group: Rats were subjected to experimental endometriosis as described above, and FM (10 mg/kg i.p. fotemustine (single dose)) was administered on the seventh day.
(3) DM group: Rats were subjected to experimental endometriosis as described above, and DM (0.1 mg/kg/day i.p. dexamethasone) was administered on the seventh day and for the next 7 days.
(4) FM+DM group: Rats were subjected to experimental endometriosis as described above, and FM+DM (10 mg/kg i.p. fotemustine (single dose) and 0.1 mg/kg/day i.p. dexamethasone) was administered on the seventh day and for the next 7 days.
(5) Sham group: Rats were injected intraperitoneally with 500 μL of PBS without endometrial tissue and the vehicle (saline) was administered.

The dose of FM+DM was based on previous experiments.

In order to evaluate endometriotic lesions, rats were sacrificed at 7 days after endometriosis induction. Laparotomy was performed to collect the endometriotic implants and further processed for molecular analysis.

### 4.4. Enzyme-Linked Immunosorbent Assay

Peritoneal fluid was collected. IL6, VEGF, IL-1β, TNF-α, IL2 and PGE2 were calculated using an ELISA kit (BioLegend, San Diego, CA, USA) [12,26].

### 4.5. Histological Examination

For histopathological assessment, endometriosis lesions and stomachs were fixed at room temperature in buffered formaldehyde solution, then sections were stained with H and E and evaluated using a Leica DM6 microscope (Leica Microsystems SpA, Milan, Italy) equipped with a motorized stage and associated with Leica LAS X Navigator software (Leica Microsystems SpA, Milan, Italy) [27,28]. Histopathologic scores were applied as described previously [14].

### 4.6. Immunohistochemical Analysis

Immunohistochemical localization of Ki67 was performed as already described [29,30]. The sections were incubated overnight with primary antibodies: anti-Ki67 antibody (1:500 in PBS *v*/*v*; Santa Cruz Biotechnology, Heidelberg, Germany, sc-23900). All sections were washed with PBS and then treated as previously reported [31,32]. Stained sections were observed using a Leica DM6 microscope following a typical procedure [33]. The histogram profile was related to the positive pixel intensity value obtained [34].

### 4.7. Western Blot Analysis

Western blots were performed as already described [35,36]. Specific primary antibodies—IκB-α (1:1000 in PBS *v*/*v*; Santa Cruz Biotechnology, sc-1643) or anti-NFκb (1:1000 in PBS *v*/*v*; Santa Cruz Biotechnology, sc-8008) or anti-COX-2 (1:500 in PBS *v*/*v*; Santa Cruz Biotechnology, sc-376861) or anti-Bcl-2 (1:1000 in PBS *v*/*v*; Santa Cruz Biotechnology, sc-7382) or anti-Bax (1:1000 in PBS *v*/*v*; Santa Cruz Biotechnology, sc-7480) or anti-proliferating nuclear antigen (PCNA) (1:500 in PBS *v*/*v*; Santa Cruz Biotechnology, sc-56) or anti-p-38 (1:1000 in PBS *v*/*v*; Santa Cruz Biotechnology, sc-166182) or anti-pERK (1:500 in PBS *v*/*v*; Santa Cruz Biotechnology, sc-7383) or anti-pJNK (1:1000 in PBS *v*/*v*; Santa Cruz Biotechnology, sc-6254)—were mixed in 5% *w/v* nonfat dried milk solution and were incubated at 4 °C overnight. Blots were incubated (Santa Cruz Biotechnology, sc-6254) with peroxidase-conjugated bovine anti-mouse IgG secondary antibody or peroxidase-conjugated goat anti-rabbit IgG (Jackson Immuno Research) for 1 h at room temperature [37,38]. To verify the equal amounts of protein, membranes were also incubated with the antibody against β-actin (Santa Cruz Biotechnology). Signals were detected with an enhanced chemiluminescence detection system reagent (Super-Signal West Pico Chemiluminescent Substrate, Pierce, Monza, Italy) [39]. The relative expression of the protein bands was quantified by densitometry with Bio-Rad ChemiDoc XRS software. Images of the blot signals were imported to analysis software (Image Quant TL, v2003) [40].

### 4.8. Terminal Deoxynucleotidyl Nick-End Labeling (TUNEL) Assay

Apoptosis was analyzed by a TUNEL assay using an in situ cell death detection kit. TUNEL staining for apoptotic cell nuclei was performed as described previously [42].

### 4.9. Statistical Evaluation

All values are expressed as the mean plus the standard error of the mean (SEM) of *N* observations. For in vivo studies, *N* represents the number of animals used. The results were analyzed by t-test, and the Kolmogorov–Smirnov test was applied to analyze the normal distribution of the data. A *p*-value of less than 0.05 was considered significant: * *p* < 0.05 vs. vehicle, ** *p* < 0.01 vs. vehicle, *** *p* < 0.001 vs. vehicle, # *p* < 0.05 vs. sham, ## *p* < 0.01 vs. sham, ### *p* < 0.001 vs. sham.

## Data Availability

The data presented in this study are available on request from the corresponding author.
